# Relationship of common variants in *MPP7*, *TIMP2* and *CASP8* genes with the risk of chronic achilles tendinopathy

**DOI:** 10.1038/s41598-019-54097-y

**Published:** 2019-11-26

**Authors:** Xin Kang, Bin Tian, Liang Zhang, Zhaogang Ge, Yang Zhao, Yingang Zhang

**Affiliations:** 1 Department of Orthopedics, the First Affiliated Hospital of Xi’an Jiao Tong University, Xi’an, Shaanxi China; 2 Department of Sports Medicine, Honghui Hospital, Xi’an Jiaotong University, Xi’an, Shaanxi China

**Keywords:** Genetic predisposition to disease, Risk factors

## Abstract

Previous etiologic studies have indicated that both environmental and genetic factors play important roles in the occurrence and development of chronic Achilles tendinopathy (AT). A recent study documented the results of the largest genome-wide association study for chronic AT to date, indicating that MPP7, TIMP2 and CASP8 may be involved in the occurrence and development of chronic AT. In this study, we aimed to investigate whether *MPP7*, *TIMP2* and *CASP8* were associated with susceptibility to chronic AP in a Han Chinese population. A total of 3,680 study subjects comprised 1,288 chronic AT cases, and 2,392 healthy controls were recruited. Forty-four tag SNPs (7 from *CASP8*, 20 from *MPP7*, and 17 from *TIMP2*) were genotyped in the study. Genetic association analyses were performed at both single marker and haplotype levels. Functional consequences of significant SNPs were examined in the RegulomeDB and GTEx databases. Two SNPs, SNP rs1937810 (OR [95%CI] = 1.20 [1.09–1.32], χ^2^ = 13.50, *P* = 0.0002) in *MPP7* and rs4789932 (OR [95%CI] = 1.24 [1.12–1.37], χ^2^ = 17.98, *P* = 2.23 × 10^−5^) in *TIMP2*, were significantly associated with chronic AT. Significant eQTL signals for SNP rs4789932 on *TIMP2* were identified in human heart and artery tissues. Our results provide further supportive evidence for the association of the *TIMP2* and *MPP7* genes with chronic AT, which supports important roles for *TIMP2* and *MPP7* in the etiology of chronic AT, adding to the current understanding of the susceptibility of chronic AT.

## Introduction

Chronic Achilles tendinopathy (AT) is a degenerative disease in both athletes and the general population^1^. Approximately 11% of the populations worldwide develop chronic AT in their lifetime^2^, which is difficult to treat and requires prolonged treatment and rehabilitation. Previous etiologic studies have indicated that environmental factors and self-diseases play important roles in the occurrence and development of chronic AT, such as age over 60 years, overuse, renal failure and diabetes mellitus^3^. Nevertheless, many case-control studies have found significant association signals between single nucleotide polymorphisms (SNPs) and chronic AT in Europeans^4–7^. Since chronic AT is a multifactorial disease with a complex genetic component, additional candidate genes should be investigated.

A recent study documented the results of the largest genome-wide association study (GWAS) for chronic AT to date, identifying borderline significant evidence of an association of rs1937810 in membrane protein palmitoylated 7 (MPP7) gene to Achilles tendon injury^8^. Moreover, this study also tested the association between previously reported SNPs and Achilles tendon injury, including *COL5A1, MMP3, TNC*, and *ADAMTS14*. However, only the rs4789932 variant in the tissue inhibitor of the metalloproteinase 2 (*TIMP2*) gene and the rs1045485 variant in the caspase-8 (CASP8) gene had moderate evidence for replication^8^. Based on the above results, MPP7, TIMP2 and CASP8 may be involved in the occurrence and development of chronic AT.

Accumulating evidence shows that the disruption of extracellular matrix (ECM) homeostasis may lead to excessive tenocyte apoptosis and eventually cause chronic AT^9,10^. Hence, genes that encode proteins with a role in maintaining the integrity of the tendon ECM and tenocyte apoptosis might associate with chronic AT. TIMP2 plays a role in inhibiting the activity of metalloproteinases, which could regulate ECM integrity. Decreasing RNA levels of TIMP2 have been demonstrated in the human degenerate Achilles tendon compared to healthy tissue^11^. In addition, serum TIMP2 protein remains high even as long as three years post-Achilles tendon injury^12^. CASP8 is an important part of the apoptosis pathway. Studies have indicated that the apoptosis pathway can induce tendon apoptosis in ECM remodeling by MMPs following tissue injury^13^. In addition, researchers have also found the upregulation of CASP8 in tendinopathy^14^. MPP7 is a CREB target and its functional mediator^15^. Previous studies have demonstrated that CREB can regulate TIMP2 in oral cancer HSC-3 cells^16^. Hence, MPP7 may regulate TIMP2 and finally influence ECM, resulting in chronic AT. Considered collectively, these data suggest that variability in chronic AT susceptibility may be related to the variants of MPP7, TIMP2 and CASP8. Although there are studies on the association between MPP7, TMIP2, CASP8 and AT, the studies only focus on Caucasians and Africans. Given of genetic heterogeneity of chronic AT in different populations, replications of the study in different populations would be desirable to validate the results. To date, no information has been available on the Han Chinese population between these genes and chronic AT. Therefore, in our study, we aimed to investigate whether the MPP7, TIMP2 and CASP8 genes were associated with susceptibility to chronic AT in a Han Chinese population.

## Methods

### Study subjects

In the study, 3,680 study subjects comprised 1,288 chronic AT cases, and 2,392 healthy individuals were collected from Honghui Hospital of Xi’an Jiaotong University between June 2014 and May 2018. These samples come from a shared sample database that needs to be authorized, and the sample size of this database is constantly expanding. Since the subjects involved in the study of Nie et al^17^. were also from this sample database, the inclusion and exclusion criteria in details for our study subjects can refer to the study of Nie *et al*.^17^. Notably, to restrict the genetic heterogeneity of the participants, all of the subjects enrolled were born in the local area. Characteristic information for our study subjects were summarized in Table 1. There were no obvious differences between both groups (cases and controls) in age, gender, smoking and alcohol drinking, but a significant difference was found in BMI. Informed consent was written by each participant. The study was carried out based on the ethical guidelines of the Declaration of Helsinki (version 2002) and was approved by the Ethics Committee of Honghui Hospital of Xi’an Jiaotong University.

**Table 1:** Characteristic information for our study subjects.

Variables	Cases (N = 1,288)	Controls (N = 2,392)	Statistics	*P*-value
Age, years	41.1 ± 8.6	40.9 ± 8.4	*T* = 0.70	0.49
BMI, kg/m^2^	25.9 ± 1.7	25.5 ± 1.7	*T* = 6.07	<0.001
Gender (%)				
Male	958 (74)	1778 (74)		
Female	330 (26)	614 (26)	χ^2^ = 7.27 × 10^−30^	1.00
Smoking (%)				
Yes	146 (11)	261 (11)		
No	1142 (89)	2131 (89)	χ^2^ = 0.11	0.74
Alcohol Drinking (%)				
Yes	312 (24)	575 (24)		
No	976 (76)	1817 (76)	χ^2^ = 0.007	0.93

### SNP selection and genotyping

Tagged SNPs with minor allele frequency (MAF) > = 0.1 in *MPP7* and *TIMP2* and MAF > = 0.05 in *CASP8* based on 1000 genome data points of Han Chinese populations were chosen for genotyping, and the *r*^2^ criterion used for tagging was 0.5 for these gene regions. A total of 44 SNPs (7 from *CASP8*, 20 from *MPP7*, and 17 from *TIMP2*) were genotyped in the study (Supplemental Table S1). All SNPs were not in the exon regions of *MPP7 TIMP2* and *CASP8* genes. Following the manufacturer’s protocol (Genomic DNA kit, Axygen Scientific, Inc., CA, USA), we extracted genomic DNA from peripheral blood leukocytes. A high-throughput Sequenom MassARRAY platform (Sequenom, San Diego, CA, USA) was utilized for SNP genotyping. Briefly, the signals from the platform were automatically analyzed by Sequenom Typer 4.0 software, and genotype data were generated from the processed results. To estimate the genotyping quality, 5% of samples were repeated for genotyping. With a concordance rate of 100%, the quality of genotyping data was confirmed. The case/control status of the samples was blinded to the technicians during the genotyping process. All SNPs had MAFs greater than 0.05 and were in Hardy-Weinberg equilibrium in our control samples (Supplemental Table S1).

### Statistical analyses

Single SNP analyses were performed with χ^2^ tests at the genotypic and allelic levels. Linkage disequilibrium (LD) blocks were estimated according to the algorithm published in the study of Gabriel *et al*.^18^, and haplotypic analyses were conducted on these LD blocks. Single SNP analyses were also stratified in gender, smoking and alcohol drinking status. In addition, to further examine the potential gene by gene interactions among the three genes, we conducted case-only analyses for multiple SNP pairs^19^. All genetic association and interaction analyses mentioned above were conducted using Plink^20^. LD plots of *CASP8*, MPP7 and *TIMP2* were made using Haploview^21^. We applied Bonferroni’s corrections to address issues of multiple comparisons. The significance threshold of the P value was 0.05/44 ≈ 0.001 in single SNP analyses.

### Bioinformatics analyses

The Function of significant SNPs were examined in RegulomeDB^22^. RegulomeDB is a public database designed for noncoding SNP annotations through integrating data from the ENCODE project and other published literature. We have also examined the association between the significant SNPs and the expression levels of their relevant genes in many human tissues in the GTEx database^23^.

## Results

### Significant genetic association signals

We identified two SNPs, SNP rs1937810 (OR [95% CI] = 1.20[1.09–1.32], χ^2^ = 13.50, *P* = 0.0002) in *MPP7* and rs4789932 (OR [95% CI] = 1.24[1.12–1.37], χ^2^ = 17.98, *P* = 2.23 × 10^−5^) in *TIMP2*, to be strongly correlated with the susceptibility to chronic AT (Table 2). Significant signals were identified at both the genotypic and allelic levels. The C alleles of both SNPs were associated with the increased risk of chronic AT. The results of single SNP analyses are presented in Supplemental Table S2. LD structures were constructed (Supplemental Figure S1, S2 and S3), and 12 LD blocks were obtained from our genetic data. All results of haplotype-based association analyses are summarized in Supplemental Table S3, which indicated the similar association pattern with single marker-based association analyses.

**Table 2:** Results of genotypic and allelic association analysis for SNP rs1937810 and rs4789932.

CHR	POS	Gene	SNP	Status	Genotypic Analyses			χ^2^	*P*	Allelic Analyses		OR[95%CI]	χ^2^	*P*
					CC	CT	TT			C	T			
10	28175021	*MPP7*	rs1937810	Cases	259	608	421			1126	1450			
				Controls	354	1172	866	17.69	0.0001	1880	2904	1.20[1.09–1.32]	13.50	0.0002
17	78928193	*TIMP2*	rs4789932	Cases	200	584	504			984	1592			
				Controls	281	1029	1082	17.42	0.0002	1591	3193	1.24[1.12–1.37]	17.98	2.23 × 10^−5^

CHR: chromosome; POS: position.

### Stratification and gene by gene interaction analyses

Stratification analyses were performed for both SNP rs1937810 and rs4789932 in gender, smoking and alcohol drinking status. The genetic effects of both SNPs were not significantly different in the stratified groups (Supplemental Table S4). Although the association signals in some stratified groups were not significant, this might be due to the limited statistical power introduced by the reduced sample size. A total of 599 SNP pairs were analyzed for gene by gene interactions among *CASP8*, MPP7 and *TIMP2*. Although 23 SNP pairs were identified as nominally significant (Supplemental Table S5), no SNP pair survived for multiple comparison corrections.

### Functional consequences of SNP rs1937810 and rs4789932

**Figure 1:**
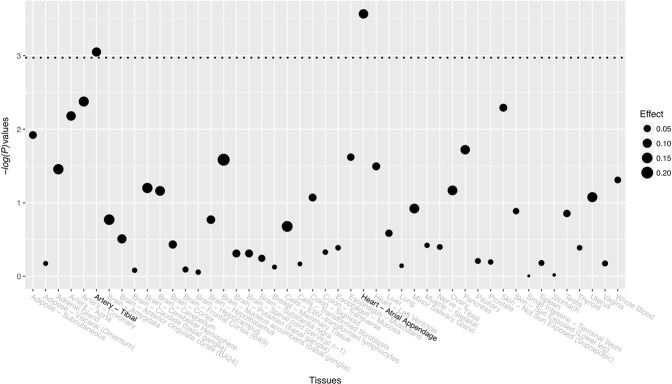
eQTL signals for SNP rs4789932 on *TIMP2*. The threshold of *P* values is indicated by a dotted line.

Since both significant SNPs were noncoding variants, they do not alter the protein sequence translated by the genes. We examined the potential functional consequences of both SNPs in RegulomeDB. RegulomeDB has a self-developed score system with a score ranging from 1–7. A higher score indicates less functional significance. SNP rs1937810 has a score of 6, and SNP rs4789932 has a score of 4. Both SNPs showed very limited functional consequences. In addition to RegulomeDB, we also examined both SNPs in the GTEx database for their eQTL patterns. No significant eQTL signals were identified for SNP rs1937810 on *MPP7* after adjusting for multiple comparisons (Supplemental Table S6). Significant eQTL signals for SNP rs4789932 on *TIMP2* were identified in human heart and artery tissues (Fig. 1 and Supplemental Table S7).

## Discussion

With the fast development and application of sequencing and genetic association analyses for studying genetic susceptibility of complex diseases, candidate gene-based association studies have successfully identified susceptibility loci for many complex diseases^24–37^. In this study, we identified two SNPs, rs1937810 in *MPP7* and rs4789932 in *TIMP2*, as significantly associated with the disease status of chronic AT in Chinese Han populations. Both SNPs have been reported in genome-wide association screens for AT conducted by Kim *et al*.^8^, and the direction of effect size of both SNPs in our study was in accordance with this previous report, which was conducted based on a mixed population mainly comprised of study subjects with European ancestry. Furthermore, the significant signals in *TIMP2* gene were also identified in Han Chinese population from the 2019 study of Nie *et al*.^17^.

Genetic markers of *CASP8* were not identified to be significantly associated with chronic AT in our samples. However, in a study performed by Kim et al., SNP rs1045485 in *CASP8* was significantly associated with chronic AT^8^. In the present study, this SNP was not analyzed because of its limited polymorphic nature in Chinese populations. Therefore, the nonsignificant signals of *CASP8* could be at least partly explained by different LD structures between Chinese Han and European populations. To investigate the contribution of *CASP8* to the risk of chronic AT in Chinese Han populations, a set of higher density markers should be selected and genotyped in the future.

Previous studies have demonstrated a potential biological connection among protein products of *CASP8*, *TIMP2* and *MPP7*^13–16^. In the present study, we examined the pair-wise gene by gene interactions. However, no significant findings were obtained. We should be careful to interpret these negative results because interaction analyses often require a larger sample size (for the same level of statistical power) compared to single marker-based association analyses. In addition, we tested 599 SNP pairs, which resulted in severe multiple comparisons. To address this problem, we applied Bonferroni’s correction, which is considered a very conservative method. Thus, in the future, a larger sample size and a better designed study are still needed to thoroughly investigate the potential epistasis patterns among the three genes.

MPP7 is a member of the Membrane-Associated Guanylate Kinase (MAGUK) subfamily of proteins, which was found in a tripartite complex with DLG1 and LIN7A or LIN7C^38^. Many studies have reported ectopic calcification in tendons in clinical samples and in animal models, which eventually leads to chronic AT with an increase in the rupture rate^39^. A previous GWAS study identified a significant association between bone mineral density (BMD) scores and MPP7^40^. Moreover, bone mass was lower in a *mpp7* knock-down zebrafish compared with the wide-type, suggesting that MPP7 is closely related to bone metabolism^41^. In addition, a case-control association study also found that MPP7 is a susceptibility gene for osteoporosis^42^. Hence, MPP7 may regulate bone formation and increase the rate of endochondral ossification, leading to chronic AT. In addition, the imbalance between matrix metalloproteinases (MMPs) and tissue inhibitors of metalloproteinases (TIMPs) leads to the excessive degradation of extracellular matrix (ECM) in chronic AT patients^43^. Among these proteins, TIMP2 is a general endogenous inhibitor of MMPs that inhibits soluble and membrane-bound MMPs^44^. Previous studies have found that patients with chronic AT showed significantly lower expression levels of TIMP2 in human degenerate AT compared to healthy tissue^45^. Additionally, aging was found to significantly reduce the expression level of TIMP2 in rabbit patellar tendons^46^. Furthermore, researchers have also found a significant mRNA expression change in TIMP2 tendons in an AT rat model^47^. Hence, TIMP2 may play an important role in tendon degradation and chronic AT because expression changes have been speculated to disrupt the TIMP/MMP balance and adversely alter ECM homeostasis. Both rs1937810 and rs4789932 were noncoding SNPs. Therefore, these SNPs cannot alter the protein structure encoded by genes. Our bioinformatics analyses showed that both SNPs had very limited functional consequences in the regulation of gene expression. In this sense, it is likely that both SNPs were just surrogates of some underlying ungenotyped variants. These variants with true effects could be common polymorphisms, as we have selected and genotyped in this present study, or they could be a set of rare or low-frequency variants that contribute to the risk of chronic AT together. As a candidate gene-based association study, we only genotyped a set of tag SNPs, and the information coverage of these SNPs might not be sufficient. In the future, sequencing-based studies should be conducted to thoroughly investigate the genetic architecture of *MPP7* and *TIMP2*.

Significant eQTL signals for SNP rs4789932 on *TIMP2* were identified from tissues of human heart and artery based on data extracted from GTEx. Nevertheless, we need to be careful to interpret these results. First, the targeted tissues for chronic AT should be tendon. Unfortunately, this type of tissue was not included in the GTEx database. Significant eQTL hits identified in human heart and artery tissues might offer us very limited information for the potential effects of this SNP on *TIMP2* in tendons and therefore might be irrelevant to the pathology of chronic AT. In addition, data from GTEx were collected from individuals with unknown status on chronic AT. A comparison of the gene expression levels of *TIMP2* in chronic AT cases and controls could be more informative from the present study. Therefore, functional studies are needed in the future to investigate the eQTL patterns of these significant SNPs on genes to which they mapped.

This present study suffered from several limitations. First, population stratifications as a potential confounder might be a problem and might introduce false positive signals. As a candidate gene-based association study, we cannot perform any statistical procedure, such as principal component analysis, to address this issue. However, in the sample recruitment process, we applied specific inclusion criteria to restrict the genetic background and heterogeneity of our study subjects. We believe that this strategy would at least partly address this problem. Another limitation is that in the present study, we do not have a replication set to replicate the significant hits. In the future, replication studies, especially those designed based on other populations, are still needed.

In summary, our results provide further supportive evidence that *TIMP2* and MPP7 contribute to the risk of chronic AT. Both SNPs rs1937810 in *MPP7* and rs4789932 in *TIMP2* may confer the risk of chronic AT and be useful in the informative assessment of the genetic risk for chronic AT susceptibility. Combined with previous findings, we provided evidence to support important roles for *TIMP2* and *MPP7* in the etiology of chronic AT, adding to the current understanding of the susceptibility of chronic AT.

## Supplementary information

Supplemental Materials
