# Supplementary material for: Relationship of common variants in MPP7, TIMP2 and CASP8 genes with the risk of chronic achilles tendinopathy
Source: Sci Rep. 2019 Nov 26;9:17627. doi: 10.1038/s41598-019-54097-y (PMC6879592; doi:10.1038/s41598-019-54097-y)
Supplement: Supplementary file 1 — Supplemental Materials [file 41598_2019_54097_MOESM1_ESM.docx]

***Title***: Relationship of common variants in *MPP7*, *TIMP2* and *CASP8* genes with the risk of chronic achilles tendinopathy

***Author names and affiliations***: Xin Kang^1,2^, Bin Tian^2^, Liang Zhang^2^, Zhaogang Ge^2^, Yang Zhao^2^, and Yingang Zhang^1^

^1^ Department of Orthopedics, the First Affiliated Hospital of Xi'an Jiao Tong University, Xi'an, Shaanxi, China;

^2^ Department of Sports Medicine, Honghui Hospital, Xi’an Jiaotong University, Xi’an Shaanxi, China.

***Corresponding Author***:

Yingang Zhang, Department of Orthopedics, the First Affiliated Hospital of Xi’an Jiaotong University, 277 Yanta West Road, Xi'an, 710061, China.

Tel: 86-29-62818386; Fax: 86-29-62818386; E-mail: dryigzhang@163.com

Supplemental Table S1. MAF and results of Hardy-Weinberg equilibrium tests for the 44 SNPs selected for genotyping.

| CHR | Gene | POS | SNP | FUNC | MAF | HWE |
| --- | --- | --- | --- | --- | --- | --- |
| 2 | *CASP8* | 201235371 | rs35392215 | intron | 0.07 | 0.24 |
| 2 | *CASP8* | 201237962 | rs3769827 | intron | 0.20 | 0.70 |
| 2 | *CASP8* | 201241269 | rs117118871 | intron | 0.06 | 0.14 |
| 2 | *CASP8* | 201246236 | rs7608692 | intron | 0.30 | 0.52 |
| 2 | *CASP8* | 201247838 | rs10192461 | intron | 0.20 | 0.61 |
| 2 | *CASP8* | 201249901 | rs12990906 | intron | 0.46 | 0.97 |
| 2 | *CASP8* | 201286440 | rs3769818 | intron | 0.29 | 0.77 |
| 10 | *MPP7* | 28063314 | rs11006820 | intron | 0.40 | 1.00 |
| 10 | *MPP7* | 28069610 | rs7919756 | intron | 0.11 | 0.54 |
| 10 | *MPP7* | 28069620 | rs2763319 | intron | 0.17 | 0.37 |
| 10 | *MPP7* | 28071734 | rs11006837 | intron | 0.13 | 0.86 |
| 10 | *MPP7* | 28079353 | rs4749300 | intron | 0.42 | 0.67 |
| 10 | *MPP7* | 28091280 | rs11006854 | intron | 0.12 | 0.70 |
| 10 | *MPP7* | 28119609 | rs10047289 | intron | 0.18 | 0.94 |
| 10 | *MPP7* | 28119827 | rs11006883 | intron | 0.48 | 0.62 |
| 10 | *MPP7* | 28124725 | rs11006887 | intron | 0.14 | 0.74 |
| 10 | *MPP7* | 28126235 | rs2490062 | intron | 0.10 | 0.51 |
| 10 | *MPP7* | 28155776 | rs2985528 | intron | 0.18 | 0.84 |
| 10 | *MPP7* | 28170770 | rs16928572 | intron | 0.12 | 0.63 |
| 10 | *MPP7* | 28175021 | rs1937810 | intron | 0.41 | 0.20 |
| 10 | *MPP7* | 28176891 | rs192414060 | intron | 0.10 | 0.44 |
| 10 | *MPP7* | 28211458 | rs113263669 | intron | 0.13 | 0.86 |
| 10 | *MPP7* | 28212416 | rs7081651 | intron | 0.40 | 0.52 |
| 10 | *MPP7* | 28212646 | rs10763652 | intron | 0.15 | 0.58 |
| 10 | *MPP7* | 28257701 | rs139574072 | intron | 0.16 | 0.28 |
| 10 | *MPP7* | 28262020 | rs1953324 | intron | 0.45 | 0.54 |
| 10 | *MPP7* | 28274292 | rs147937433 | intron | 0.11 | 0.76 |
| 17 | *TIMP2* | 78857255 | rs3786146 | intron | 0.22 | 0.59 |
| 17 | *TIMP2* | 78860177 | rs9916809 | intron | 0.12 | 0.62 |
| 17 | *TIMP2* | 78860571 | rs16971783 | intron | 0.11 | 0.67 |
| 17 | *TIMP2* | 78867780 | rs9909541 | intron | 0.19 | 0.64 |
| 17 | *TIMP2* | 78869991 | rs71368041 | intron | 0.18 | 0.78 |
| 17 | *TIMP2* | 78870403 | rs56019347 | intron | 0.19 | 0.55 |
| 17 | *TIMP2* | 78870425 | rs200829956 | intron | 0.20 | 0.17 |
| 17 | *TIMP2* | 78880991 | rs8065599 | intron | 0.44 | 0.38 |
| 17 | *TIMP2* | 78882547 | rs7502935 | intron | 0.34 | 0.89 |
| 17 | *TIMP2* | 78888398 | rs9901429 | intron | 0.21 | 0.15 |
| 17 | *TIMP2* | 78901057 | rs4789864 | intron | 0.35 | 0.62 |
| 17 | *TIMP2* | 78914309 | rs8065768 | intron | 0.36 | 0.48 |
| 17 | *TIMP2* | 78918084 | rs535153737 | intron | 0.17 | 0.20 |
| 17 | *TIMP2* | 78918986 | rs200229500 | intron | 0.26 | 0.83 |
| 17 | *TIMP2* | 78925708 | rs8179091 | near-gene-5' | 0.44 | 0.45 |
| 17 | *TIMP2* | 78925807 | rs8179090 | near-gene-5' | 0.18 | 0.53 |
| 17 | *TIMP2* | 78928193 | rs4789932 | near-gene-5' | 0.35 | 0.13 |

CHR: chromosome; POS: position; FUNC: function; HWE: *P* values for Hardy-Weinberg equilibrium tests.

Supplemental Table S2. Full results of single marker based association analyses.

| CHR | SNP | POS | A1 | F_A | F_U | A2 | χ^2^ | *P* | OR | L95 | U95 |
| --- | --- | --- | --- | --- | --- | --- | --- | --- | --- | --- | --- |
| 2 | rs35392215 | 201235371 | G | 0.07 | 0.07 | C | 0.28 | 0.59 | 1.05 | 0.87 | 1.27 |
| 2 | rs3769827 | 201237962 | G | 0.20 | 0.20 | A | 0.25 | 0.62 | 0.97 | 0.86 | 1.09 |
| 2 | rs117118871 | 201241269 | T | 0.06 | 0.06 | G | 0.48 | 0.49 | 1.07 | 0.88 | 1.31 |
| 2 | rs7608692 | 201246236 | A | 0.30 | 0.29 | G | 0.65 | 0.42 | 1.04 | 0.94 | 1.16 |
| 2 | rs10192461 | 201247838 | A | 0.21 | 0.20 | G | 0.34 | 0.56 | 1.04 | 0.92 | 1.17 |
| 2 | rs12990906 | 201249901 | C | 0.46 | 0.46 | T | 0.19 | 0.66 | 0.98 | 0.89 | 1.08 |
| 2 | rs3769818 | 201286440 | A | 0.29 | 0.29 | G | 0.24 | 0.62 | 0.97 | 0.88 | 1.08 |
| 10 | rs11006820 | 28063314 | T | 0.40 | 0.40 | G | 0.05 | 0.82 | 0.99 | 0.90 | 1.09 |
| 10 | rs7919756 | 28069610 | C | 0.11 | 0.11 | A | 0.50 | 0.48 | 0.95 | 0.81 | 1.10 |
| 10 | rs2763319 | 28069620 | C | 0.17 | 0.17 | A | 0.52 | 0.47 | 1.05 | 0.92 | 1.19 |
| 10 | rs11006837 | 28071734 | T | 0.14 | 0.13 | A | 0.29 | 0.59 | 1.04 | 0.90 | 1.20 |
| 10 | rs4749300 | 28079353 | C | 0.43 | 0.42 | A | 0.20 | 0.65 | 1.02 | 0.93 | 1.13 |
| 10 | rs11006854 | 28091280 | C | 0.13 | 0.12 | T | 0.53 | 0.47 | 1.06 | 0.91 | 1.22 |
| 10 | rs10047289 | 28119609 | C | 0.18 | 0.18 | T | 0.12 | 0.73 | 0.98 | 0.86 | 1.11 |
| 10 | rs11006883 | 28119827 | A | 0.49 | 0.48 | C | 0.17 | 0.68 | 1.02 | 0.93 | 1.12 |
| 10 | rs11006887 | 28124725 | T | 0.15 | 0.14 | C | 0.48 | 0.49 | 1.05 | 0.92 | 1.20 |
| 10 | rs2490062 | 28126235 | A | 0.10 | 0.10 | T | 0.18 | 0.67 | 0.97 | 0.82 | 1.13 |
| 10 | rs2985528 | 28155776 | A | 0.19 | 0.18 | C | 0.39 | 0.53 | 1.04 | 0.92 | 1.18 |
| 10 | rs16928572 | 28170770 | G | 0.12 | 0.12 | A | 0.44 | 0.51 | 1.05 | 0.91 | 1.22 |
| 10 | rs1937810 | 28175021 | C | 0.44 | 0.39 | T | 13.50 | 2.39E-04 | 1.20 | 1.09 | 1.32 |
| 10 | rs192414060 | 28176891 | A | 0.11 | 0.10 | G | 0.30 | 0.58 | 1.04 | 0.89 | 1.22 |
| 10 | rs113263669 | 28211458 | G | 0.13 | 0.13 | T | 0.27 | 0.61 | 0.96 | 0.84 | 1.11 |
| 10 | rs7081651 | 28212416 | T | 0.40 | 0.39 | C | 0.17 | 0.68 | 1.02 | 0.93 | 1.13 |
| 10 | rs10763652 | 28212646 | C | 0.16 | 0.15 | T | 0.40 | 0.53 | 1.04 | 0.91 | 1.19 |
| 10 | rs139574072 | 28257701 | G | 0.16 | 0.16 | T | 0.34 | 0.56 | 1.04 | 0.91 | 1.18 |
| 10 | rs1953324 | 28262020 | G | 0.46 | 0.45 | A | 0.15 | 0.70 | 1.02 | 0.93 | 1.12 |
| 10 | rs147937433 | 28274292 | T | 0.12 | 0.11 | C | 0.65 | 0.42 | 1.06 | 0.92 | 1.24 |
| 17 | rs3786146 | 78857255 | C | 0.22 | 0.22 | T | 3.84E-03 | 0.95 | 1.00 | 0.89 | 1.13 |
| 17 | rs9916809 | 78860177 | A | 0.12 | 0.12 | C | 2.44E-03 | 0.96 | 1.00 | 0.86 | 1.17 |
| 17 | rs16971783 | 78860571 | A | 0.11 | 0.11 | T | 0.24 | 0.62 | 0.96 | 0.82 | 1.12 |
| 17 | rs9909541 | 78867780 | T | 0.19 | 0.19 | C | 0.00 | 0.98 | 1.00 | 0.89 | 1.13 |
| 17 | rs71368041 | 78869991 | C | 0.17 | 0.18 | A | 0.35 | 0.56 | 0.96 | 0.85 | 1.09 |
| 17 | rs56019347 | 78870403 | A | 0.20 | 0.19 | G | 0.11 | 0.74 | 1.02 | 0.90 | 1.15 |
| 17 | rs200829956 | 78870425 | A | 0.20 | 0.19 | G | 0.34 | 0.56 | 1.04 | 0.92 | 1.17 |
| 17 | rs8065599 | 78880991 | T | 0.44 | 0.44 | C | 0.04 | 0.85 | 1.01 | 0.92 | 1.11 |
| 17 | rs7502935 | 78882547 | A | 0.33 | 0.34 | G | 0.39 | 0.53 | 0.97 | 0.87 | 1.07 |
| 17 | rs9901429 | 78888398 | G | 0.21 | 0.21 | A | 0.19 | 0.66 | 1.03 | 0.91 | 1.15 |
| 17 | rs4789864 | 78901057 | T | 0.35 | 0.35 | C | 0.09 | 0.77 | 1.02 | 0.92 | 1.12 |
| 17 | rs8065768 | 78914309 | T | 0.35 | 0.36 | C | 0.27 | 0.61 | 0.97 | 0.88 | 1.08 |
| 17 | rs535153737 | 78918084 | T | 0.17 | 0.17 | A | 0.04 | 0.84 | 1.01 | 0.89 | 1.15 |
| 17 | rs200229500 | 78918986 | A | 0.26 | 0.26 | C | 0.64 | 0.42 | 0.96 | 0.86 | 1.07 |
| 17 | rs8179091 | 78925708 | A | 0.44 | 0.44 | G | 0.01 | 0.94 | 1.00 | 0.90 | 1.10 |
| 17 | rs8179090 | 78925807 | G | 0.18 | 0.18 | C | 0.18 | 0.67 | 1.03 | 0.91 | 1.16 |
| 17 | rs4789932 | 78928193 | C | 0.38 | 0.33 | T | 17.98 | 2.23E-05 | 1.24 | 1.12 | 1.37 |

CHR: chromosome; POS: position; A1: minor allele; F_A: minor allele frequency in cases; F_U: minor allele frequency in controls; A2: major allele; L95: lower bound of 95% confidence interval; U95: upper bound of 95% confidence interval.

Supplemental Table S3. Full results of haplotypic association analysis.

| Gene | Haplotype | F_A | F_U | χ^2^ | DF | *P* | SNPs |
| --- | --- | --- | --- | --- | --- | --- | --- |
| *CASP8* | OMNIBUS | NA | NA | 0.72 | 2 | 0.6975 | rs10192461\|rs12990906 |
| *CASP8* | AC | 0.20 | 0.20 | 0.20 | 1 | 0.6581 | rs10192461\|rs12990906 |
| *CASP8* | GC | 0.25 | 0.26 | 0.68 | 1 | 0.4102 | rs10192461\|rs12990906 |
| *CASP8* | GT | 0.54 | 0.54 | 0.14 | 1 | 0.7122 | rs10192461\|rs12990906 |
| *MPP7* | OMNIBUS | NA | NA | 0.76 | 2 | 0.6828 | rs11006820\|rs7919756 |
| *MPP7* | TC | 0.11 | 0.11 | 0.72 | 1 | 0.3951 | rs11006820\|rs7919756 |
| *MPP7* | TA | 0.30 | 0.29 | 0.15 | 1 | 0.6982 | rs11006820\|rs7919756 |
| *MPP7* | GA | 0.60 | 0.60 | 0.03 | 1 | 0.8557 | rs11006820\|rs7919756 |
| *MPP7* | OMNIBUS | NA | NA | 0.28 | 2 | 0.8673 | rs11006837\|rs4749300 |
| *MPP7* | TC | 0.13 | 0.13 | 0.13 | 1 | 0.7220 | rs11006837\|rs4749300 |
| *MPP7* | AC | 0.29 | 0.29 | 0.09 | 1 | 0.7673 | rs11006837\|rs4749300 |
| *MPP7* | AA | 0.57 | 0.58 | 0.27 | 1 | 0.6060 | rs11006837\|rs4749300 |
| *MPP7* | OMNIBUS | NA | NA | 1.98 | 3 | 0.5772 | rs11006854\|rs10047289\|rs11006883 |
| *MPP7* | CCA | 0.12 | 0.12 | 0.10 | 1 | 0.7478 | rs11006854\|rs10047289\|rs11006883 |
| *MPP7* | TCA | 0.05 | 0.06 | 1.34 | 1 | 0.2463 | rs11006854\|rs10047289\|rs11006883 |
| *MPP7* | TTA | 0.32 | 0.31 | 0.74 | 1 | 0.3904 | rs11006854\|rs10047289\|rs11006883 |
| *MPP7* | TTC | 0.51 | 0.52 | 0.22 | 1 | 0.6374 | rs11006854\|rs10047289\|rs11006883 |
| *MPP7* | OMNIBUS | NA | NA | 15.58 | 2 | 0.0004 | rs1937810\|rs192414060 |
| *MPP7* | CA | 0.10 | 0.10 | 0.12 | 1 | 0.7283 | rs1937810\|rs192414060 |
| *MPP7* | CG | 0.34 | 0.29 | 14.09 | 1 | 0.0002 | rs1937810\|rs192414060 |
| *MPP7* | TG | 0.56 | 0.61 | 13.96 | 1 | 0.0002 | rs1937810\|rs192414060 |
| *MPP7* | OMNIBUS | NA | NA | 0.26 | 2 | 0.8797 | rs139574072\|rs1953324 |
| *MPP7* | GG | 0.16 | 0.16 | 0.16 | 1 | 0.6909 | rs139574072\|rs1953324 |
| *MPP7* | TG | 0.30 | 0.30 | 0.04 | 1 | 0.8504 | rs139574072\|rs1953324 |
| *MPP7* | TA | 0.54 | 0.55 | 0.22 | 1 | 0.6428 | rs139574072\|rs1953324 |
| *TIMP2* | OMNIBUS | NA | NA | 0.14 | 2 | 0.9302 | rs3786146\|rs9916809 |
| *TIMP2* | CA | 0.11 | 0.11 | 0.04 | 1 | 0.8449 | rs3786146\|rs9916809 |
| *TIMP2* | CC | 0.11 | 0.11 | 0.12 | 1 | 0.7277 | rs3786146\|rs9916809 |
| *TIMP2* | TC | 0.78 | 0.78 | 0.01 | 1 | 0.9133 | rs3786146\|rs9916809 |
| *TIMP2* | OMNIBUS | NA | NA | 0.12 | 2 | 0.9401 | rs200829956\|rs8065599 |
| *TIMP2* | AT | 0.20 | 0.19 | 0.06 | 1 | 0.8068 | rs200829956\|rs8065599 |
| *TIMP2* | GT | 0.25 | 0.24 | 0.03 | 1 | 0.8620 | rs200829956\|rs8065599 |
| *TIMP2* | GC | 0.56 | 0.56 | 0.12 | 1 | 0.7297 | rs200829956\|rs8065599 |
| *TIMP2* | OMNIBUS | NA | NA | 1.62 | 2 | 0.4460 | rs7502935\|rs9901429 |
| *TIMP2* | AG | 0.21 | 0.21 | 0.13 | 1 | 0.7199 | rs7502935\|rs9901429 |
| *TIMP2* | AA | 0.12 | 0.13 | 1.60 | 1 | 0.2055 | rs7502935\|rs9901429 |
| *TIMP2* | GA | 0.67 | 0.66 | 0.35 | 1 | 0.5522 | rs7502935\|rs9901429 |
| *TIMP2* | OMNIBUS | NA | NA | 4.26 | 2 | 0.1186 | rs4789864\|rs8065768 |
| *TIMP2* | TT | 0.35 | 0.35 | 0.00 | 1 | 0.9588 | rs4789864\|rs8065768 |
| *TIMP2* | CT | 0.01 | 0.01 | 4.25 | 1 | 0.0392 | rs4789864\|rs8065768 |
| *TIMP2* | CC | 0.64 | 0.64 | 0.15 | 1 | 0.7007 | rs4789864\|rs8065768 |
| *TIMP2* | OMNIBUS | NA | NA | 1.41 | 2 | 0.4938 | rs535153737\|rs200229500 |
| *TIMP2* | TA | 0.17 | 0.17 | 0.00 | 1 | 0.9782 | rs535153737\|rs200229500 |
| *TIMP2* | AA | 0.09 | 0.09 | 1.39 | 1 | 0.2383 | rs535153737\|rs200229500 |
| *TIMP2* | AC | 0.74 | 0.74 | 0.56 | 1 | 0.4531 | rs535153737\|rs200229500 |
| *TIMP2* | OMNIBUS | NA | NA | 26.92 | 2 | 1.43E-06 | rs8179090\|rs4789932 |
| *TIMP2* | GC | 0.18 | 0.18 | 0.15 | 1 | 0.6977 | rs8179090\|rs4789932 |
| *TIMP2* | CC | 0.20 | 0.16 | 24.73 | 1 | 6.59E-07 | rs8179090\|rs4789932 |
| *TIMP2* | CT | 0.62 | 0.67 | 18.11 | 1 | 2.09E-05 | rs8179090\|rs4789932 |

F_A: haplotypic frequency in cases; F_U: haplotypic frequency in controls; DF: degree of freedom.

Supplemental Table S4. Genetic association analyses stratified by gender, smoking and alcohol drinking status.

| CHR | SNP | POS | A1 | A2 | Groups | F_A | F_U | χ^2^ | *P* | OR | L95 | U95 |
| --- | --- | --- | --- | --- | --- | --- | --- | --- | --- | --- | --- | --- |
| 17 | rs4789932 | 78928193 | C | T | Gender-Male | 0.38 | 0.34 | 9.93 | 0.0016 | 1.20 | 1.07 | 1.35 |
|  |  |  |  |  | Gender-Female | 0.39 | 0.32 | 9.05 | 0.0026 | 1.35 | 1.11 | 1.65 |
|  |  |  |  |  | Smoking-Yes | 0.38 | 0.34 | 1.29 | 0.2570 | 1.19 | 0.88 | 1.60 |
|  |  |  |  |  | Smoking-No | 0.38 | 0.33 | 16.78 | 4.19E-05 | 1.25 | 1.12 | 1.39 |
|  |  |  |  |  | Alcohol Drinking-Yes | 0.39 | 0.33 | 5.32 | 0.0211 | 1.27 | 1.04 | 1.55 |
|  |  |  |  |  | Alcohol Drinking-No | 0.38 | 0.33 | 12.72 | 0.0004 | 1.23 | 1.10 | 1.38 |
| 10 | rs1937810 | 28175021 | C | T | Gender-Male | 0.43 | 0.40 | 7.22 | 0.0072 | 1.17 | 1.04 | 1.31 |
|  |  |  |  |  | Gender-Female | 0.45 | 0.38 | 7.20 | 0.0073 | 1.30 | 1.07 | 1.58 |
|  |  |  |  |  | Smoking-Yes | 0.41 | 0.40 | 0.12 | 0.7275 | 1.05 | 0.79 | 1.41 |
|  |  |  |  |  | Smoking-No | 0.44 | 0.39 | 14.26 | 0.0002 | 1.22 | 1.10 | 1.35 |
|  |  |  |  |  | Alcohol Drinking-Yes | 0.40 | 0.40 | 0.11 | 0.7364 | 1.04 | 0.85 | 1.26 |
|  |  |  |  |  | Alcohol Drinking-No | 0.45 | 0.39 | 16.22 | 0.0001 | 1.26 | 1.12 | 1.41 |

CHR: chromosome; POS: position; A1: minor allele; A2: major allele. F_A: minor allele frequency in cases; F_U: minor allele frequency in controls; L95: lower bound of 95% confidence interval; U95: upper bound of 95% confidence interval.

Supplemental Table S5. SNP pairs achieved nominal significance in case-only genetic interaction analyses.

| CHR1 | SNP1 | CHR2 | SNP2 | STAT | *P* |
| --- | --- | --- | --- | --- | --- |
| 2 | rs117118871 | 17 | rs8179090 | 12.26 | 0.0005 |
| 10 | rs147937433 | 17 | rs9916809 | 8.49 | 0.0036 |
| 2 | rs35392215 | 17 | rs4789932 | 7.30 | 0.0069 |
| 2 | rs117118871 | 10 | rs1937810 | 7.04 | 0.0080 |
| 2 | rs117118871 | 10 | rs11006820 | 6.94 | 0.0084 |
| 2 | rs117118871 | 10 | rs4749300 | 6.79 | 0.0092 |
| 10 | rs10763652 | 17 | rs9909541 | 5.96 | 0.0146 |
| 2 | rs117118871 | 10 | rs7081651 | 5.85 | 0.0156 |
| 10 | rs1937810 | 17 | rs9909541 | 5.79 | 0.0161 |
| 2 | rs35392215 | 10 | rs11006854 | 5.58 | 0.0182 |
| 2 | rs117118871 | 17 | rs8179091 | 5.06 | 0.0245 |
| 2 | rs7608692 | 10 | rs113263669 | 5.02 | 0.0250 |
| 10 | rs10763652 | 17 | rs71368041 | 4.94 | 0.0263 |
| 2 | rs117118871 | 10 | rs2985528 | 4.73 | 0.0297 |
| 2 | rs117118871 | 10 | rs2490062 | 4.60 | 0.0320 |
| 2 | rs3769827 | 10 | rs7081651 | 4.52 | 0.0336 |
| 2 | rs12990906 | 10 | rs10763652 | 4.42 | 0.0355 |
| 10 | rs113263669 | 17 | rs71368041 | 4.41 | 0.0358 |
| 10 | rs2985528 | 17 | rs535153737 | 4.33 | 0.0374 |
| 10 | rs10763652 | 17 | rs4789932 | 4.07 | 0.0437 |
| 2 | rs12990906 | 10 | rs139574072 | 4.01 | 0.0453 |
| 2 | rs7608692 | 17 | rs16971783 | 3.99 | 0.0458 |
| 2 | rs10192461 | 10 | rs139574072 | 3.97 | 0.0463 |

Supplemental Table S6. eQTL signals of SNP rs1937810 on *MPP7* from multiple human tissues.

| Gene | SNP | *P*-Value | NES | T-statistic | Tissue |
| --- | --- | --- | --- | --- | --- |
| *MPP7* | rs1937810 | 0.02 | 0.16 | 2.30 | Heart - Atrial Appendage |
| *MPP7* | rs1937810 | 0.02 | -0.13 | -2.30 | Adipose - Visceral (Omentum) |
| *MPP7* | rs1937810 | 0.08 | -0.08 | -1.80 | Artery - Aorta |
| *MPP7* | rs1937810 | 0.08 | 0.13 | 1.80 | Brain - Caudate (basal ganglia) |
| *MPP7* | rs1937810 | 0.08 | 0.16 | 1.80 | Brain - Frontal Cortex (BA9) |
| *MPP7* | rs1937810 | 0.12 | -0.11 | -1.50 | Cells - Transformed fibroblasts |
| *MPP7* | rs1937810 | 0.15 | -0.05 | -1.50 | Esophagus - Mucosa |
| *MPP7* | rs1937810 | 0.16 | 0.24 | 1.40 | Brain - Substantia nigra |
| *MPP7* | rs1937810 | 0.19 | 0.05 | 1.30 | Adipose - Subcutaneous |
| *MPP7* | rs1937810 | 0.21 | 0.21 | 1.30 | Brain - Cerebellum |
| *MPP7* | rs1937810 | 0.21 | -0.04 | -1.30 | Esophagus - Muscularis |
| *MPP7* | rs1937810 | 0.22 | -0.04 | -1.20 | Skin - Not Sun Exposed (Suprapubic) |
| *MPP7* | rs1937810 | 0.25 | -0.06 | -1.20 | Artery - Coronary |
| *MPP7* | rs1937810 | 0.26 | 0.16 | 1.10 | Uterus |
| *MPP7* | rs1937810 | 0.31 | 0.05 | 1.00 | Breast - Mammary Tissue |
| *MPP7* | rs1937810 | 0.33 | 0.09 | 0.99 | Brain - Cortex |
| *MPP7* | rs1937810 | 0.33 | 0.04 | 0.98 | Testis |
| *MPP7* | rs1937810 | 0.37 | -0.03 | -0.89 | Whole Blood |
| *MPP7* | rs1937810 | 0.38 | 0.09 | 0.87 | Adrenal Gland |
| *MPP7* | rs1937810 | 0.41 | 0.07 | 0.83 | Pituitary |
| *MPP7* | rs1937810 | 0.44 | -0.04 | -0.77 | Muscle - Skeletal |
| *MPP7* | rs1937810 | 0.49 | 0.02 | 0.68 | Artery - Tibial |
| *MPP7* | rs1937810 | 0.53 | 0.08 | 0.63 | Brain - Amygdala |
| *MPP7* | rs1937810 | 0.56 | -0.05 | -0.59 | Minor Salivary Gland |
| *MPP7* | rs1937810 | 0.61 | 0.04 | 0.50 | Pancreas |
| *MPP7* | rs1937810 | 0.62 | -0.07 | -0.50 | Brain - Cerebellar Hemisphere |
| *MPP7* | rs1937810 | 0.62 | -0.01 | -0.49 | Skin - Sun Exposed (Lower leg) |
| *MPP7* | rs1937810 | 0.64 | -0.05 | -0.47 | Ovary |
| *MPP7* | rs1937810 | 0.66 | -0.02 | -0.44 | Lung |
| *MPP7* | rs1937810 | 0.67 | 0.03 | 0.43 | Colon - Sigmoid |
| *MPP7* | rs1937810 | 0.68 | 0.03 | 0.41 | Brain - Putamen (basal ganglia) |
| *MPP7* | rs1937810 | 0.68 | -0.05 | -0.42 | Brain - Spinal cord (cervical c-1) |
| *MPP7* | rs1937810 | 0.73 | 0.02 | 0.34 | Colon - Transverse |
| *MPP7* | rs1937810 | 0.74 | -0.05 | -0.33 | Cells - EBV-transformed lymphocytes |
| *MPP7* | rs1937810 | 0.76 | 0.02 | 0.30 | Vagina |
| *MPP7* | rs1937810 | 0.81 | 0.02 | 0.23 | Brain - Anterior cingulate cortex (BA24) |
| *MPP7* | rs1937810 | 0.81 | -0.02 | -0.24 | Brain - Nucleus accumbens (basal ganglia) |
| *MPP7* | rs1937810 | 0.81 | 0.01 | 0.24 | Small Intestine - Terminal Ileum |
| *MPP7* | rs1937810 | 0.82 | -0.01 | -0.23 | Stomach |
| *MPP7* | rs1937810 | 0.84 | 0.02 | 0.20 | Brain - Hippocampus |
| *MPP7* | rs1937810 | 0.85 | -0.03 | -0.19 | Liver |
| *MPP7* | rs1937810 | 0.88 | 0.01 | 0.15 | Heart - Left Ventricle |
| *MPP7* | rs1937810 | 0.91 | 0.01 | 0.11 | Thyroid |
| *MPP7* | rs1937810 | 0.92 | -0.01 | -0.09 | Prostate |
| *MPP7* | rs1937810 | 0.94 | 0.01 | 0.07 | Brain - Hypothalamus |
| *MPP7* | rs1937810 | 0.95 | -0.0032 | -0.06 | Nerve - Tibial |
| *MPP7* | rs1937810 | 0.99 | 0.0008 | 0.01 | Spleen |

NES: normalized effect size.

Supplemental Table S7. eQTL signals of SNP rs4789932 on *TIMP2* from multiple human tissues.

| Gene | SNP | *P*-Value | NES | T-statistic | Tissue |
| --- | --- | --- | --- | --- | --- |
| *TIMP2* | rs4789932 | 0.0003 | -0.11 | -3.70 | Heart - Atrial Appendage |
| *TIMP2* | rs4789932 | 0.0009 | -0.10 | -3.40 | Artery - Tibial |
| *TIMP2* | rs4789932 | 0.0042 | -0.13 | -2.90 | Artery - Coronary |
| *TIMP2* | rs4789932 | 0.01 | -0.06 | -2.80 | Skin - Not Sun Exposed (Suprapubic) |
| *TIMP2* | rs4789932 | 0.01 | -0.11 | -2.70 | Artery - Aorta |
| *TIMP2* | rs4789932 | 0.01 | -0.06 | -2.50 | Adipose - Subcutaneous |
| *TIMP2* | rs4789932 | 0.02 | -0.11 | -2.40 | Pancreas |
| *TIMP2* | rs4789932 | 0.02 | -0.05 | -2.30 | Esophagus - Muscularis |
| *TIMP2* | rs4789932 | 0.03 | 0.21 | 2.30 | Brain - Nucleus accumbens (basal ganglia) |
| *TIMP2* | rs4789932 | 0.03 | -0.07 | -2.20 | Heart - Left Ventricle |
| *TIMP2* | rs4789932 | 0.04 | 0.14 | 2.10 | Adrenal Gland |
| *TIMP2* | rs4789932 | 0.05 | -0.04 | -2.00 | Whole Blood |
| *TIMP2* | rs4789932 | 0.06 | -0.14 | -1.90 | Brain - Cerebellar Hemisphere |
| *TIMP2* | rs4789932 | 0.07 | 0.12 | 1.80 | Ovary |
| *TIMP2* | rs4789932 | 0.07 | 0.12 | 1.80 | Brain - Cerebellum |
| *TIMP2* | rs4789932 | 0.08 | -0.13 | -1.70 | Uterus |
| *TIMP2* | rs4789932 | 0.09 | -0.06 | -1.70 | Colon - Sigmoid |
| *TIMP2* | rs4789932 | 0.12 | -0.12 | -1.60 | Minor Salivary Gland |
| *TIMP2* | rs4789932 | 0.13 | -0.03 | -1.50 | Skin - Sun Exposed (Lower leg) |
| *TIMP2* | rs4789932 | 0.14 | 0.05 | 1.50 | Testis |
| *TIMP2* | rs4789932 | 0.17 | 0.15 | 1.40 | Brain - Amygdala |
| *TIMP2* | rs4789932 | 0.17 | 0.08 | 1.40 | Brain - Hypothalamus |
| *TIMP2* | rs4789932 | 0.21 | -0.16 | -1.30 | Cells - EBV-transformed lymphocytes |
| *TIMP2* | rs4789932 | 0.26 | -0.05 | -1.10 | Liver |
| *TIMP2* | rs4789932 | 0.31 | -0.10 | -1.00 | Brain - Anterior cingulate cortex (BA24) |
| *TIMP2* | rs4789932 | 0.37 | -0.07 | -0.89 | Brain - Cortex |
| *TIMP2* | rs4789932 | 0.38 | -0.02 | -0.88 | Muscle - Skeletal |
| *TIMP2* | rs4789932 | 0.40 | -0.02 | -0.84 | Nerve - Tibial |
| *TIMP2* | rs4789932 | 0.41 | 0.02 | 0.82 | Esophagus - Mucosa |
| *TIMP2* | rs4789932 | 0.41 | 0.02 | 0.82 | Thyroid |
| *TIMP2* | rs4789932 | 0.47 | -0.02 | -0.72 | Colon - Transverse |
| *TIMP2* | rs4789932 | 0.49 | 0.07 | 0.69 | Brain - Putamen (basal ganglia) |
| *TIMP2* | rs4789932 | 0.49 | 0.07 | 0.70 | Brain - Spinal cord (cervical c-1) |
| *TIMP2* | rs4789932 | 0.57 | 0.05 | 0.57 | Brain - Substantia nigra |
| *TIMP2* | rs4789932 | 0.62 | -0.02 | -0.49 | Pituitary |
| *TIMP2* | rs4789932 | 0.64 | 0.02 | 0.47 | Prostate |
| *TIMP2* | rs4789932 | 0.66 | 0.02 | 0.45 | Spleen |
| *TIMP2* | rs4789932 | 0.67 | -0.01 | -0.43 | Adipose - Visceral (Omentum) |
| *TIMP2* | rs4789932 | 0.67 | -0.03 | -0.43 | Vagina |
| *TIMP2* | rs4789932 | 0.68 | 0.01 | 0.41 | Cells - Transformed fibroblasts |
| *TIMP2* | rs4789932 | 0.72 | -0.01 | -0.36 | Lung |
| *TIMP2* | rs4789932 | 0.75 | -0.01 | -0.31 | Breast - Mammary Tissue |
| *TIMP2* | rs4789932 | 0.81 | -0.03 | -0.24 | Brain - Frontal Cortex (BA9) |
| *TIMP2* | rs4789932 | 0.83 | 0.02 | 0.21 | Brain - Caudate (basal ganglia) |
| *TIMP2* | rs4789932 | 0.88 | -0.02 | -0.16 | Brain - Hippocampus |
| *TIMP2* | rs4789932 | 0.96 | -0.0013 | -0.05 | Stomach |
| *TIMP2* | rs4789932 | 0.99 | -0.0005 | -0.01 | Small Intestine - Terminal Ileum |

NES: normalized effect size.


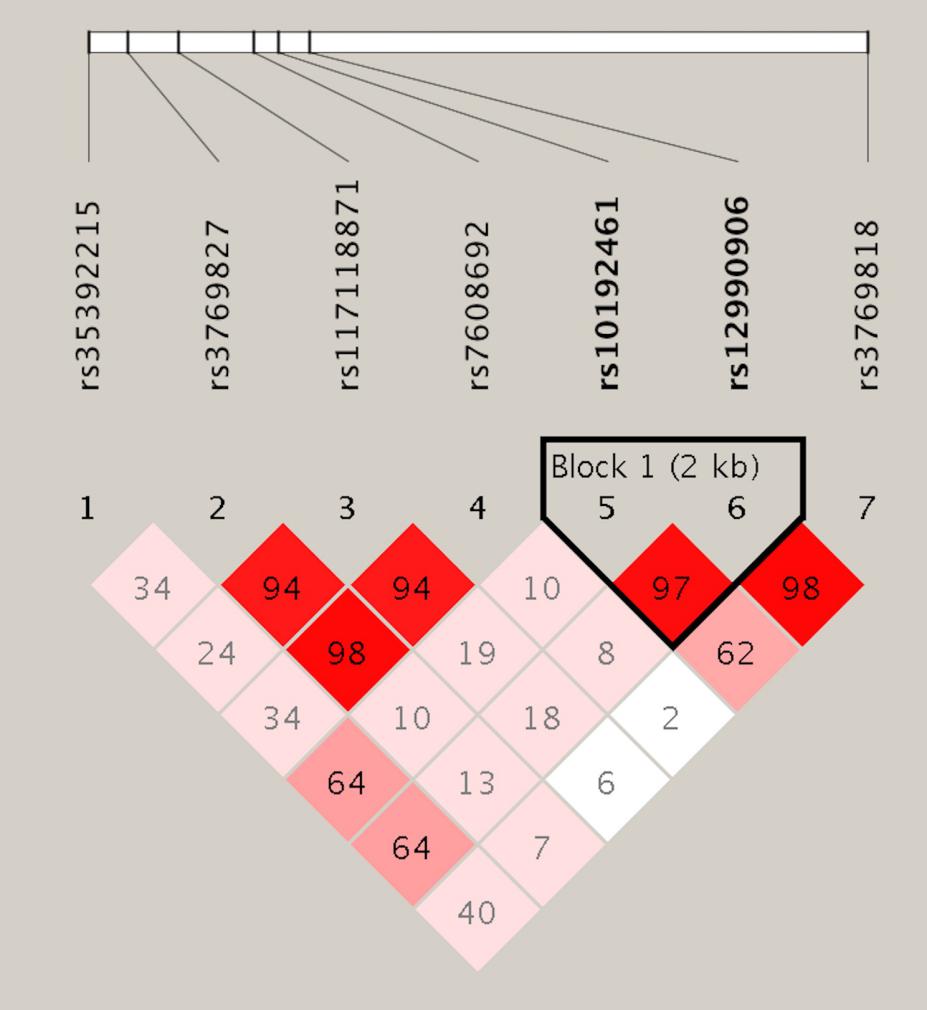


Supplemental Figure S1. LD structure of SNPs genotyped in gene *CASP8*. Values of D’ were indicated in each cells.


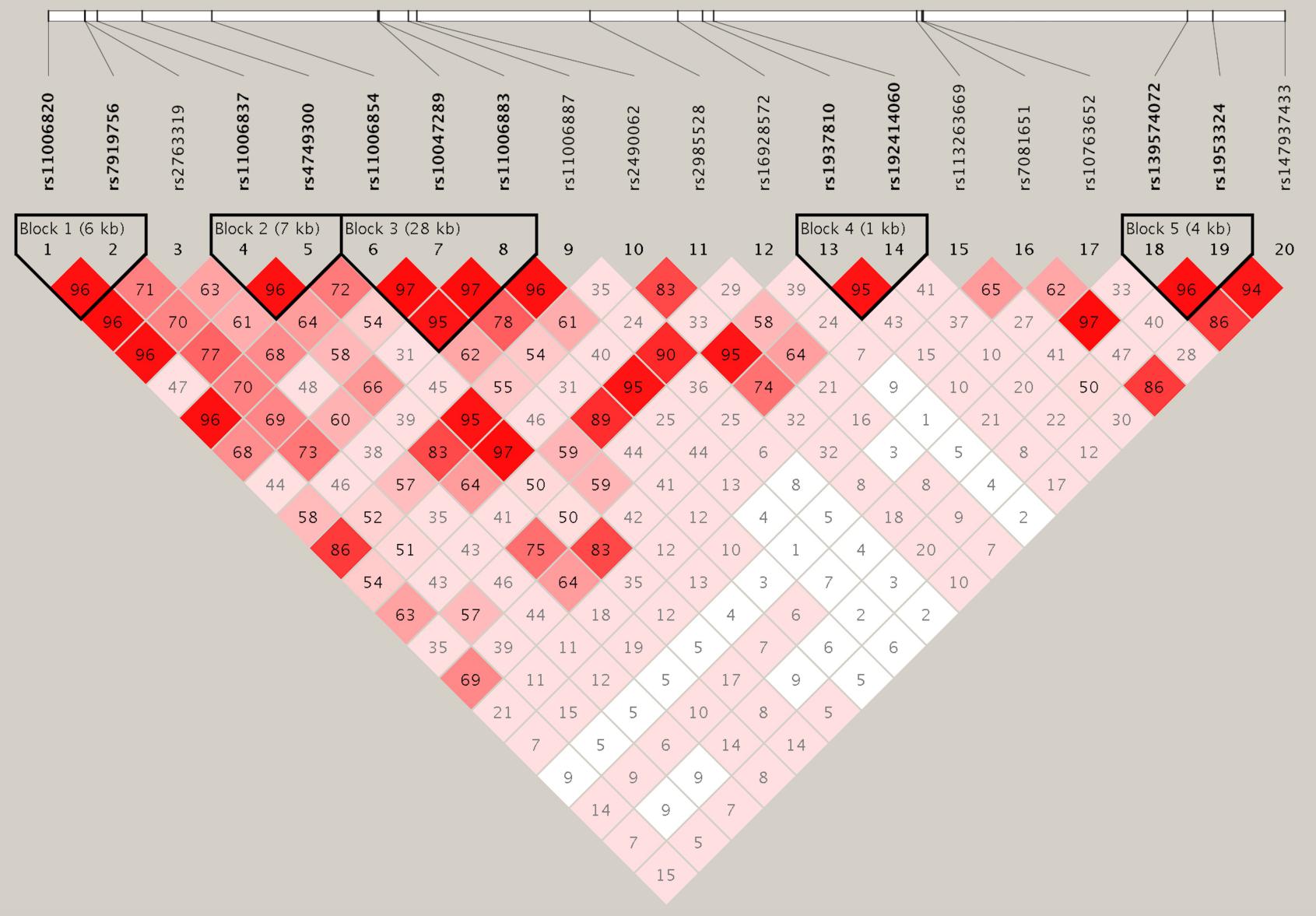


Supplemental Figure S2. LD structure of SNPs genotyped in gene *MPP7*. Values of D’were indicated in each cells.


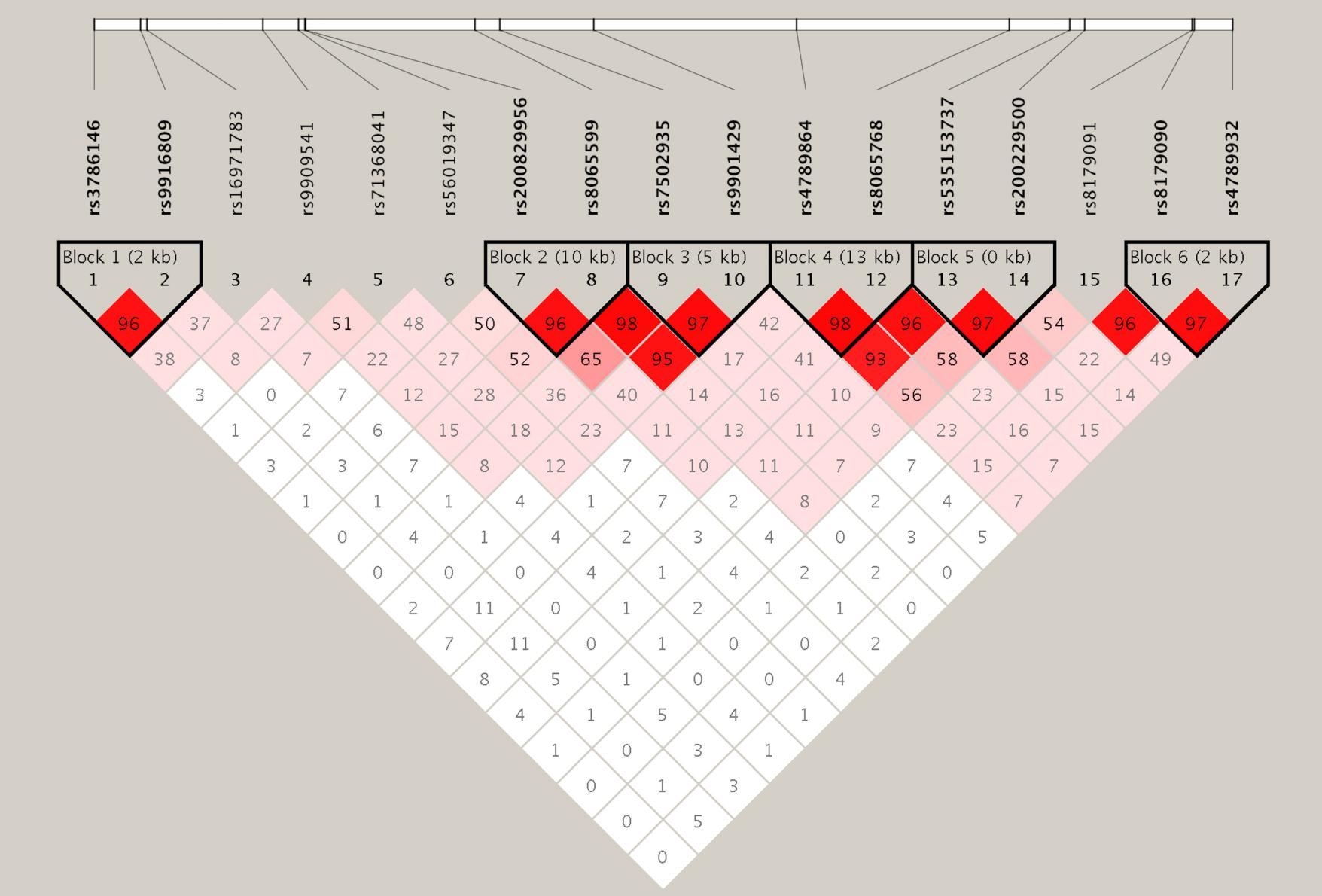


Supplemental Figure S3. LD structure of SNPs genotyped in gene *TIMP2*. Values of D’ were indicated in each cells.
